# Correction: Hannotier et al. A CFO-Assisted Algorithm for Wireless Time-Difference-of-Arrival Localization Networks: Analytical Study and Experimental Results. *Sensors* 2024, *24*, 737

**DOI:** 10.3390/s25113563

**Published:** 2025-06-05

**Authors:** Cédric Hannotier, François Horlin, François Quitin

**Affiliations:** Brussels School of Engineering, Université libre de Bruxelles, Avenue Franklin Roosevelt 50, 1000 Brussels, Belgium; francois.horlin@ulb.be (F.H.); francois.quitin@ulb.be (F.Q.)

## Figure Correction

In the original publication [1], there was a mistake in Figure 10 as published. The corrected Figure 10 appears below.


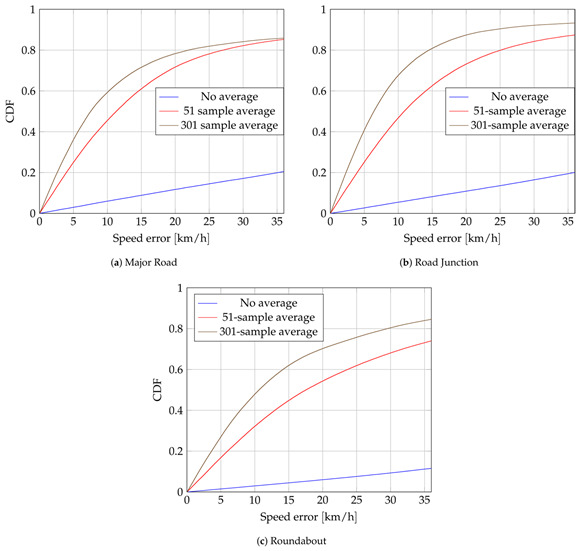


## Text Correction

1. In the original Section 3.3, the sentence “Figure 10 shows the eCDFs of the speed error for each scenario” has been updated to read “Figure 10 shows the eCDFs of the speed error for each scenario, defined as $\left|∥v_{T}∥-∥\hat{v_{T}}∥\right|$, where $v_{T}$ is the true velocity, $\hat{v_{T}}$ the estimated one”.

2. In Section 3.3, Paragraphs 2–3 have been updated as follows:

Lowering $\sigma_{\Delta \mathrm{CFO}}$ increases the accuracy of the speed estimation. However, targets are not moving faster than 55 km/h and, 95% of the time, they do not reach 30 km/h. Hence, getting accurate speed estimations—relative to the target’s low speed—is challenging. eCDFs of the errors of direction, defined as the angle between actual and estimated velocities, are shown in Figure 11. This illustrates that the coarse estimation of the direction of the target is possible. It performs worse in roundabout scenarios. Apart from the higher $\sigma_{\Delta \mathrm{CFO}}$, averaging over several measurements is less effective since it averages measurements with different directions.

The authors apologize for any inconvenience caused and state that the scientific conclusions are unaffected. The original article has been updated. This correction was approved by the Academic Editor. The original publication has also been updated.
